# Mixture and Exponential Arcs on Generalized Statistical Manifold

**DOI:** 10.3390/e20030147

**Published:** 2018-02-25

**Authors:** Luiza H. F. de Andrade, Francisca L. J. Vieira, Rui F. Vigelis, Charles C. Cavalcante

**Affiliations:** 1 Department of Natural Science, Mathematics and Statistics, Federal Rural University of Semi-Arid Region, Mossoró 59.625-900, Brazil; 2 Department of Mathematics, Regional University of Cariri, Juazeiro do Norte 63105-000, Brazil; 3 Computer Engineering, Campus Sobral, Federal University of Ceará, Sobral 62010-560, Brazil; 4 Department of Teleinformatics Engineering, Federal University of Ceará, Fortaleza 60455-900, Brazil

**Keywords:** statistical manifold, *φ*-family, information geometry, exponential arcs, mixture arcs

## Abstract

In this paper, we investigate the mixture arc on generalized statistical manifolds. We ensure that the generalization of the mixture arc is well defined and we are able to provide a generalization of the open exponential arc and its properties. We consider the model of a φ-family of distributions to describe our general statistical model.

## 1. Introduction

In the geometry of statistical models, information geometry [1,2,3] is the part of probability theory dedicated to investigate probability density functions equipped with differential geometry structure. A differential-geometric structure to the multi-parameter families of distributions was provided in [4]. In the mid-1980s, other topics related to the subject, such as fiber bundle theory and duality of connections of statistical models, were investigated by Amari [5] and Amari and Nagaoka [6], respectively. In the parametric case, exponential, mixture and α-connections, as well as their dual structure, are among the most important geometric objects [6], since the dual structure of the α-connections is the key point distinguishing statistical manifolds against arbitrary differential manifolds. Divergence function is an essential topic in information geometry, for both, parametric and non-parametric cases, since a metric and dual connections can be induced from a divergence [7,8,9,10]. To find an information-geometrical foundation for multi-parameter families of probability distributions, with a more general description, is one of topics of interest in information geometry [11,12,13,14]

Non-parametric statistical models [15] are important in a wide range of areas [16,17]. In the parametric case, the manifold of probability density functions obtains a Euclidian topology from the space of its natural parameters. As for the non-parameter case, a major challenge is to define a convenient topology and a notion of convergence. Pistone and Sempi [18] were the first to formulate a rigorous infinite dimensional extension. In that work, the set $P_{\mu }$ of all strictly positive probability densities was endowed with a structure of exponential Banach manifolds, using Orlicz spaces associated to a Young function. In a later work [19], more properties of the statistical manifold were studied, specifically regarding the orthogonality condition.

Similar to in the parametric case, in non-parametric models, the mixture and exponential connections are among the most important geometric objects. To find these connections, it is necessary to guarantee the existence of the open arcs, which are the geodesics of the manifold. Using the notion of exponential convergence, Gibilisco and Pistone [20] investigated those connections. In that work, the exponential and mixture connections were built in a way that the relation between them is the same as in the parametric case. Another approach was used in [21] where the mixture arc was additionally studied. Moreover, Grasselli [21] proved that two probability densities in the same neighborhood are connected by an open mixture arc if and only if the difference between their random variables is bounded.

The exponential statistical manifold was later studied in [22], with another system of charts, the statistical model E(p), called the maximal exponential model. Cena and Pistone [22] proved that this model is the set of all positive densities connected to a given positive density *p* by an open exponential arc and viceversa. In that work, it was used the open mixture arc and the open exponential arc to discuss properties of this model as e-connection and m-connection in the same way that in [6]. This exponential model E(p) with the open exponential and mixture arcs were also studied recently by Santacroce et al., 2016 [23] and Santacroce et al., 2017 [24], where a proof of duality properties of statistical models was provided. Examples of applications of non-parametric information geometry to statistical physics using the connection by open arcs were studied in [25].

The generalization of the exponential statistical manifold has been an active topic of research in the last years. Pistone [26] used the Kaniadaki’s κ-exponential [27] in the construction of a statistical manifold. Vigelis and Cavalcante [28] proposed a φ-family of probability distributions $F_{c}^{\varphi }$, which generalizes the exponential family E(p). This generalization is based on the replacement of the exponential function by a deformed exponential φ which satisfies some properties and provides to the set $P_{\mu }$ a Banach manifold structure, so called generalized statistical manifold. In [29], a review of nonparametric information geometry with specific issues of the infinite dimensional setting is provided. In that work, the deformed exponential manifold was studied with a deformed exponential function defined in [30] and a model space was built according to the proposal in [28].

In [31] were given necessary and sufficient conditions for any two probability distributions being connected by a φ-arc. In this work, we ensure the existence of a generalized mixture arc for probability distributions in the same φ-family $F_{c}^{\varphi }$, with a deformed exponential function which satisfies some properties. Moreover, we find a generalization of open exponential arcs and we prove, in the same way that in [22], that the φ-family $F_{c}^{\varphi }$ is the component connected to a given positive density p=φ(c) and viceversa.

The rest of the paper is organized as follows. In Section 2, we revisit results about Musielak–Orlicz space and φ-family of probability distributions. We also briefly recall about the subdifferential of a convex function. In Section 3, where we provide our main results, we ensure that the generalized mixture arc is well-defined. In Section 4, we discuss the generalized, exponential and mixture arcs. Finally, our conclusions and perspectives are stated in Section 5.

## 2. Preliminary Results

The statistical manifold $P_{\mu }$ can be equipped with a structure of $C^{\infty }$-Banach Manifold, using the Musielak–Orlicz space $L^{\Phi }$ associated to the Musielak–Orlicz function $\Phi_{c}\left(t,u\right)=\varphi \left(t,c\left(t\right)+u\left(t\right)\right)-\varphi \left(t,c\left(t\right)\right)$. Each connected component of the statistical manifold gives rise to a φ-family of probability distributions $F_{c}^{\varphi }$. In this section, we provide an introduction of Musielak–Orlicz spaces and the construction of the φ-family of probability distributions.

### 2.1. φ-Families of Probability Distributions

Let (T,Σ,μ) be a σ-finite, non-atomic measure space. A function Φ:T×[0,∞)→[0,∞] is said to be a Musielak–Orlicz function if

(i) Φ(t,·) is convex and lower semi-continuous for μ-a.e. (almost everywhere) t∈T,
(ii) $\Phi \left(t,0\right)=\lim_{u↓0},\Phi \left(t,u\right)=0$ and $\lim_{u\to \infty }\Phi \left(t,u\right)=\infty$ for μ-a.e. t∈T,
(iii) Φ(·,u) is measurable for each u≥0.

We notice that Φ(t,·), by (i)-(ii), is not equal to 0 or *∞* on the interval (0,∞).

Let $L^{0}$ be the linear space of all real-value, measurable functions on *T*. Given a Musielak–Orlicz function Φ, we denote the functional $I_{\Phi }\left(u\right)=\int_{T}\Phi \left(t,|u\left(t\right)|\right)d\mu$, for any $u\in L^{0}$. The Musielak–Orlicz space, Musielak–Orlicz class, Morse–Transue space generated by a Musielak–Orlicz function Φ are defined, respectively, by

$$L^{\Phi }=\left\{u\in L^{0}:\;I_{\Phi }\left(\lambda u\right)<\infty \;\mathrm{for}\;\mathrm{some}\;\lambda >0\right\}$$

$${\tilde{L}}^{\Phi }=\left\{u\in L^{0}:\;I_{\Phi }\left(u\right)<\infty \right\},$$

and

$$E^{\Phi }=\left\{u\in L^{0}:I_{\Phi }\left(\lambda u\right)<\infty \;\mathrm{for}\;\mathrm{all}\;\lambda >0\right\}.$$

The Musielak–Orlicz space $L^{\Phi }$ is a Banach space when it is equipped with the Luxemburg norm given by

$${∥u∥}_{\Phi }=\inf\;\left\{\lambda >0:\;I_{\Phi }\left(\frac{u}{\lambda }\right)\leq 1\right\},$$

or the Orlicz norm, represented as

$${∥u∥}_{\Phi ,0}=\sup\left\{\left|\int_{T}uv\;d\mu \right|\;:\;v\in {\tilde{L}}^{\Phi^{*}}\;\mathrm{and}\;I_{\Phi^{*}}\left(v\right)\leq 1\right\},$$

where $\Phi^{*}\left(t,v\right)=\sup_{u\geq 0}\left(uv-\Phi \left(t,u\right)\right)$ is the Fenchel conjugate of Φ(t,·), which is also a Musielak–Orlicz function. These norms are equivalent and the inequalities ${∥u∥}_{\Phi }\leq {∥u∥}_{\Phi ,0}\leq 2{∥u∥}_{\Phi }$ hold for all $u\in L^{\Phi }$ [32]. A Musielak–Orlicz function is said to satisfy the $\Delta_{2}$-condition, or belong to the $\Delta_{2}$-class (denoted by $\Phi \in \Delta_{2}$), if we can find a constant α>0 and a non-negative function $f\in {\tilde{L}}^{\Phi }$ such that

(1) $$\alpha \Phi \left(t,u\right)\leq \Phi \left(t,\frac{1}{2}u\right),\;\mathrm{for}\;\mathrm{all}\;u\geq f\left(t\right),\;\mathrm{and}\;\mu -a.e.\;t\in T.$$

If the Musielak–Orlicz function Φ satisfies the $\Delta_{2}$-condition, then $I_{\Phi }\left(u\right)<\infty$ for every $u\in L^{\Phi }$ [32]. In this case $L^{\Phi }$, ${\tilde{L}}^{\Phi }$ and $E^{\Phi }$ are equal as sets. Moreover, if the Musielak–Orlicz function Φ does not satisfy the $\Delta_{2}$-condition, $E^{\Phi }$ is a proper subspace of $L^{\Phi }$. Every function Φ that satisfies the $\Delta_{2}$-condition is finite-value. Indeed, we define

(2) $$b_{\Phi }\left(t\right)=\sup\left\{u\geq 0:\Phi \left(t,u\right)<\infty \right\},$$

and assuming that $b_{\Phi }\left(t\right)<\infty$, we get $\Phi \left(t,\frac{1}{2}u\right)<\alpha \Phi \left(t,u\right)=\infty$ for all $b_{\Phi }\left(t\right)<u<2b_{\Phi }\left(t\right)$ which implies that Φ cannot satisfy the $\Delta_{2}$-condition. For more information see for instance [32,33].

We say that a Musielak–Orlicz function Φ satisfies the $\nabla_{2}$-condition, or belongs to $\nabla_{2}$-class, if we can find a constant γ>1, and a non-negative function $f\in {\tilde{L}}^{\Phi }$ such that

(3) $$\gamma \Phi \left(t,u\right)\leq \Phi \left(t,\frac{1}{2}\gamma u\right),\;\mathrm{for}\;\mathrm{all}\;u>f\left(t\right).$$

We notice that, if $\Phi \in \nabla_{2}$, then

$$d_{\Phi }=\lim_{u\to \infty }\frac{\Phi (t,u)}{u}=\lim_{u\to \infty }\Phi_{-}^{\prime }\left(t,u\right)=\lim_{u\to \infty }\Phi_{+}^{\prime }\left(t,u\right)=\infty .$$

**Example** **1.** *The function Φ:[0,∞)→[0,∞) defined by:*

Φ(u)=exp(u)−u−1

*satisfies the $\nabla_{2}$-condition and does not satisfy the $\Delta_{2}$-condition.*

The (topological) dual space of $L^{\Phi }$, is denoted by ${\left(L^{\Phi }\right)}^{*}$ and represented in the following way [32,34,35]

$${\left(L^{\Phi }\right)}^{*}=L^{\Phi^{*}}⊕{\left(L^{\Phi }\right)}_{s}^{∼},$$

where $L^{\Phi^{*}}$ is the set of the order continuous functionals and ${\left(L^{\Phi }\right)}_{s}^{∼}$ is formed by singular components. If the Musielak–Orlicz function $\Phi_{c}\in \Delta_{2}$ then all functionals in ${\left(L^{\Phi }\right)}^{*}$ are order continuous and represented by

(4) $$f_{v^{*}}\left(u\right):=\int_{T}uv^{*}d\mu ,\;\;\;\mathrm{for}\;\mathrm{all}\;u\in L^{\Phi }.$$

Otherwise, if $\Phi \notin \Delta_{2}$, then the functionals *f* in ${\left(L^{\Phi }\right)}^{*}$ can be uniquely expressed as

$$f=f_{c}+f_{s},$$

where $f_{c}$ is the order continuous component and $f_{s}$ is the singular component.

While exponential families are based on the exponential function, φ-families are based on deformed exponential functions. A deformed exponential φ:T×R→(0,∞) is a function that satisfies the following properties, for μ-a.e. t∈T [28]:

(i) φ(·) is convex and injective;
(ii) $\lim_{u\to -\infty }\varphi \left(u\right)=0$ and $\lim_{u\to \infty }\varphi \left(u\right)=\infty$;
(iii) There exists a measurable function $u_{0}:T\to \left(0,\infty \right)$ such that
  $$\int_{T}\varphi \left(c+\lambda u_{0}\right)d\mu <\infty ,\;\mathrm{for}\;\mathrm{all}\;\lambda >0,$$

for every measurable function c:T→R for which $\int_{T}\varphi \left(c\right)d\mu =1$.

In de Souza et al. [36], Lemma 1, it was shown that the constraint $\int_{T}\varphi \left(c\right)d\mu =1$ can be replaced by $\int_{T}\varphi \left(c\right)d\mu <\infty$. Thus, the condition (iii) can be rewritten as:

(iii’) There exists a measurable function $u_{0}:T\to \left(0,\infty \right)$ such that
  $$\int_{T}\varphi \left(c+\lambda u_{0}\right)d\mu <\infty ,\;\mathrm{for}\;\mathrm{all}\;\lambda >0,$$

for every measurable function c:T→R for which $\int_{T}\varphi \left(c\right)d\mu <\infty$.

There are many examples of deformed exponential functions. An example of relevance is the exponential function φ(x)=exp(x) that satisfies (i)-(iii) with $u_{0}=1_{T}$. Another example is Kaniadakis’ κ-exponential [26,27,28]:

**Example** **2.** *The Kaniadakis’ κ-exponential $\exp_{\kappa }:R\to \left(0,\infty \right)$ for κ∈[−1,1] is defined as*

$$\exp_{\kappa }\left(u\right)=\left\{\begin{array}{cc} {\left(\kappa u+\sqrt{1+\kappa^{2}u^{2}}\right)}^{\frac{1}{\kappa }}, & if\;\kappa \neq 0,\\ \exp(u) & if\;\kappa =0. \end{array}\right.$$

*The inverse of $\exp_{\kappa }$ is the Kaniadakis’ κ-logarithm*

$$\ln_{\kappa }\left(u\right)=\left\{\begin{array}{cc} \frac{u^{\kappa }-u^{-\kappa }}{2\kappa }, & if\;\kappa \neq 0,\\ \ln(u) & if\;\kappa =0. \end{array}\right.$$

One can easily notice the κ-exponential satisfies (i)–(iii) [28,36].

The Musielak–Orlicz function

(5) $$\Phi_{c}\left(t,u\right)=\varphi \left(t,c\left(t\right)+u\right)-\varphi \left(t,c\left(t\right)\right)$$

for a measurable function c:T→R such that φ(t,c(t)) is μ-integrable, was defined in [28]. Thus, the sets $L^{\Phi_{c}}$, ${\tilde{L}}^{\Phi_{c}}$ and $E^{\Phi_{c}}$ are denoted by $L_{c}^{\varphi }$, ${\tilde{L}}_{c}^{\varphi }$ and $E_{c}^{\varphi }$, respectively, when $\Phi_{c}$ is given by (5). Let

$$P_{\mu }=\left\{p\in L^{0}:p>0\;\mathrm{and}\;\int_{T}pd\mu =1\right\}$$

be the collection whose φ-family is a subset, where $L^{0}$ is the linear space of all real-valued. For each probability density $p\in P_{\mu }$, we have a φ-family of probability density associated, $F_{c}^{\varphi }=\varphi_{c}\left(B_{c}^{\varphi }\right)\subset P_{\mu }$ according to

(6) $$\varphi_{c}\left(u\right)=\varphi \left(c+u-\psi \left(u\right)u_{0}\right),\;\;\mathrm{for}\;\mathrm{each}\;u\in B_{c}^{\varphi },$$

where the set $B_{c}^{\varphi }$ is the intersection of the convex set

$$K_{c}^{\varphi }=\left\{u\in L_{c}^{\varphi }:\int_{T}\varphi \left(c+\lambda u\right)d\mu <\infty \mathrm{for}\;\mathrm{some}\;\lambda >1\right\},$$

with the closed subspace

(7) $$B_{c}^{\varphi }=\left\{u\in L_{c}^{\varphi }:\int_{T}u\varphi_{+}^{\prime }\left(c\right)d\mu =0\right\},$$

that is $B_{c}^{\varphi }=K_{c}^{\varphi }\cap B_{c}^{\varphi }$. The normalizing function $\psi :B_{c}^{\varphi }\to \left[0,\infty \right)$ is introduced so that expression (6) is a probability distribution in $P_{\mu }$. Suppose that the Musielak–Orlicz function $\Phi_{c}$ does not satisfy the $\Delta_{2}$-condition, we have that the boundary of $B_{c}^{\varphi }$, the set $\partial B_{c}^{\varphi }$, is not empty. A function $u\in B_{c}^{\varphi }$ belongs to $\partial B_{c}^{\varphi }$ if only if $\int_{T}\varphi \left(c+\lambda u\right)d\mu <\infty$ for all λ∈(0,1), and $\int_{T}\varphi \left(c+\lambda u\right)d\mu =\infty$ for each λ>1. The behavior of the normalizing function near the boundary was studied in [33,37].

It is shown that the normalizing function $\psi :K_{c}^{\varphi }\to R$ is a convex function [28]. Assuming that φ is continuously differentiable, the normalizing function is Gâteaux-differentiable and the expression for Gâteaux-derivative is

(8) $$\partial \psi \left(u\right)v=\frac{\int_{T}v\varphi^{\prime }\left(c+u-\psi \left(u\right)u_{0}\right)d\mu }{\int_{T}u_{0}\varphi^{\prime }\left(c+u-\psi \left(u\right)u_{0}\right)d\mu },$$

with $u\in K_{c}^{\varphi }$ and $v\in L_{c}^{\varphi }$.

In the next section, we recall some differentiability properties of convex functions on infinite dimensional spaces.

### 2.2. The Subdifferential of a Convex function

In this section, we discuss some properties of extended real-valued convex functions in Banach spaces, i.e., functions with values in R∪{±∞}. Mainly, we recall subdifferentials of lower semicontinuous convex functions and its properties.

Let *E* be a Banach space. A function *f* is a convex function on *E*, with the epigraph [38]

$$\mathrm{epi}\;f=\left\{(x,\alpha ):x\in E,\;\alpha \in R,\;\alpha \geq f(x)\right\}.$$

If f(x)>−∞ for every *x* and f(x)<+∞ for at least one value of *x*, we call *f* a proper function. The set

$$\mathrm{dom}\;f=\left\{x\in E:f(x)<\infty \right\}$$

denotes the effective domain of *f*. A function f:E→(−∞,∞] is said to be lower semicontinuous (l.s.c.) if for every λ∈R the set

[f≤λ]={x∈E:f(x)≤λ}

is closed.

Let $E^{*}$ be the dual space of *E*. A vector $x^{*}\in E^{*}$ is said to be a subgradient of *f* at x∈E if

$$\left(x^{*},z\right)\leq f\left(x+z\right)-f\left(x\right)\;\;\;\mathrm{for}\;\mathrm{all}\;z\in E.$$

We denote by ∂f(x) the set of subgradients of *f* at *x* and the subdifferential of *f* is the multivalued mapping x↦∂f(x) from *E* to $E^{*}$. By definition, ∂f(x) is always a closed convex subset of $E^{*}$ for each *x*. Suppose *f* is a convex function finite at *x*. One has $x^{*}\in \partial f\left(x\right)$ if and only if

$$\left(z,x^{*}\right)\leq f^{\prime }\left(x;z\right),\;\;\;\forall z\in E,$$

where

$$f^{\prime }\left(x;z\right)=\lim_{t\to 0^{+}}\frac{f(x+tz)-f(x)}{t}$$

is the directional derivative of *f* at *x* in direction z∈E. The subdifferential may be empty at points of domf, so we denote by

$$D\left(\partial f\right)=\left\{x\in E:\partial f(x)\neq \emptyset \right\},$$

the domain of ∂f and we have that D(∂f)⊂domf. We say that *f* is subdifferentiable at *x* for all x∈D(∂f).

Let *f* be a lower semicontinuous proper convex function, then intdomf⊂D(∂f) [39] (Corollary 2.38). The conjugate of *f* is the function $f^{*}:E^{*}\to \bar{R}$ defined by

(9) $$f^{*}\left(x^{*}\right)=\sup\left\{\left(x,x^{*}\right)-f\left(x\right):x\in E\right\},\;\;\;x^{*}\in E^{*}.$$

Observe that, if *f* is proper, then “sup” in Equation (9) may be restricted to the points x∈domf. The conjugate $f^{*}$ is a convex and lower semicontinuous function on $E^{*}$ and jointly with *f* satisfy the well known Young’s inequality

(10) $$\left(x,x^{*}\right)\leq f\left(x\right)+f^{*}\left(x^{*}\right),$$

with equality holding if and only if $x^{*}\in \partial f\left(x\right)$. If *f* is a lower semicontinuous function, the subdifferential $\partial f^{*}$ of the conjugate function $f^{*}$ coincides with ${\left(\partial f\right)}^{-1}$ ([39], Proposition 2.33).

It is known that, if *f* is a lower semicontinuous proper convex function, then

intdomf⊂D(∂f)⊂domf,

and it was shown in [40] that D(∂f) is, in fact, dense in domf.

**Fact** **1** (([41], Corollary 2.19), ([42], Corollary 7.2.3))**.** *Suppose x∈D(∂f). Then x∈intdomf if only if ∂f is locally bounded at x.*

**Fact** **2** (([41], Lemma 2.20), ([42], Lemma 7.2.4))**.** *If intdomf≠∅ and x∈D(∂f)∖intdomf, then ∂f(x) is unbounded.*

The subdifferential of a convex function is closely related to Gâteaux-gradient. If the convex function *f* is Gâteaux-differentiable in $x_{0}\in E$, then $\partial f(x_{0})$ consists of a single element $x^{*}=\mathrm{grad}\;f\left(x_{0}\right)$ ([39], Proposition 2.40), where gradf(x) is the Gâteaux-gradient of *f* at *x*.

In the next section, we investigate the subdifferential of the normalizing function ψ. This result will be useful for us to prove that the generalized mixture arc is well defined, which is one of our main goals in this work.

## 3. Construction of Generalized Mixture Arcs

The normalizing function $\psi :K_{c}^{\varphi }\to R$ is convex and Gâteaux-differentiable and this derivative is given by Equation (8). Hence, with these facts in mind, we can provide the expression for the generalized mixture arc as given by:

(11) $$p\left(t\right)=F^{-1}\left(\left(1-t\right)F\left(p\right)+tF\left(q\right)\right),$$

where

(12) $$F\left(p\right)=\frac{\varphi^{\prime }\left(\varphi^{-1}\left(p\right)\right)}{\int_{T}u_{0}\varphi^{\prime }\left(\varphi^{-1}\left(p\right)\right)d\mu },$$

and p,q belong to a φ-family $F_{c}^{\varphi }$. We can rewrite the functional F(p) as

(13) $$\frac{\varphi^{\prime }\left(c+u-\psi \left(u\right)u_{0}\right)}{\int_{T}u_{0}\varphi^{\prime }\left(c+u-\psi \left(u\right)u_{0}\right)d\mu },\;u\in B_{c}^{\varphi },$$

with $p=\varphi_{c}\left(c+u-\psi \left(u\right)u_{0}\right)$ and Equation (13) is the Gâteaux-gradient of ψ. Thus, for the generalized mixture arc to be well defined, it is necessary that the set of these functionals in Equation (13) be convex. As mentioned in Section 2.2, the subdifferential and Gâteaux-gradient are closely related. For this reason, we investigate the subdifferential of ψ.

### 3.1. Subdifferential of the Normalizing Function ψ

Considering that the Musielak–Orlicz function (5) does not satisfy the $\Delta_{2}$-condition, then we have that $\partial B_{c}^{\varphi }$ is not-empty [33]. The effective domain of the normalizing ψ, the set $\mathrm{dom}\;\psi =\left\{u\in B_{c}^{\varphi }:\psi \left(u\right)<\infty \right\}$ is

(14) $$\mathrm{dom}\;\psi =B_{c}^{\varphi }\cup {\left\{\partial B_{c}^{\varphi }\right\}}_{<\infty }\;,$$

where ${\left\{\partial B_{c}^{\varphi }\right\}}_{<\infty }$ is the set of points in the boundary of $B_{c}^{\varphi }$ such that ψ(u)<∞. The behavior of the normalizing function ψ near the boundary of $B_{c}^{\varphi }$ was discussed in [33]. We need to know the subdifferentials of ψ. Hence, we have to prove some properties of ψ, then we have our first result.

**Proposition** **1.** The normalizing function $\psi :B_{c}^{\varphi }\to R\cup \left\{\infty \right\}$ is lower semicontinuous.

**Proof.**  Given α∈R, let $C_{\alpha }$ be the set $C_{\alpha }=\left\{u\in B_{c}^{\varphi }:\psi \left(u\right)\leq \alpha \right\}$. To prove the statement, it suffices to show that $C_{\alpha }$ is closed. We define a set

$$B=\left\{u\in B_{c}^{\varphi }:\int_{T}\varphi \left(c+u-\alpha u_{0}\right)d\mu \leq 1\right\},$$

and we are going to prove that *B* is a closed set and that $B=C_{\alpha }$. Let $\left\{u_{n}\right\}$ be a sequence which belongs to *B*, such that $∥u_{n}-u{∥}_{\Phi_{c}}\to 0$. This way, $u_{n}\to u$, μ-a.e. Since φ is a continuous function, we have that $\varphi \left(c+u_{n}-\alpha u_{0}\right)\to \varphi \left(c+u+\alpha u_{0}\right)$, μ-a.e. From Fatou’s Lemma, it follows that

$$\begin{array}{cc} \int_{T}\varphi \left(c+u-\alpha u_{0}\right)d\mu & =\int_{T}\underset{n\to \infty }{lim inf}\varphi \left(c+u_{n}-\alpha u_{0}\right)d\mu \\ & \leq \underset{n\to \infty }{lim inf}\int_{T}\varphi \left(c+u_{n}-\alpha u_{0}\right)d\mu \\ & \leq 1 \end{array}$$

thus, u∈B and *B* is a closed set. Now, we prove that $B=C_{\alpha }$. Let *u* be a function which belongs to $C_{\alpha }$, then ψ(u)≤α. The function φ is a strictly increasing function, so that

$$\int_{T}\varphi \left(c+u-\alpha u_{0}\right)d\mu \leq \int_{T}\varphi \left(c+u-\psi \left(u\right)u_{0}\right)d\mu =1,$$

thus, u∈B.

Suppose that there exists $w\in B\setminus C_{\alpha }$, then w∈B, which implies that $\int_{T}\varphi \left(c+w-\alpha u_{0}\right)d\mu \leq 1$ and $w\notin C_{\alpha }$, which implies that ψ(w)>α. Then

$$\int_{T}\varphi \left(c+w-\alpha u_{0}\right)d\mu >\int_{T}\varphi \left(c+w-\psi \left(w\right)u_{0}\right)d\mu =1,$$

thus $\int_{T}\varphi \left(c+w-\alpha u_{0}\right)d\mu >1$. This contradicts the assumption that w∈B. Therefore, $B=C_{\alpha }$ and $C_{\alpha }$ is closed. ☐

The subdifferential of ψ at a function u∈domψ is the set

(15) $$\partial \psi \left(u\right)=\left\{u^{*}\in {\left(L^{\Phi_{c}}\right)}^{*}:\int_{T}u^{*}vd\mu \leq \psi \left(u+v\right)-\psi \left(u\right),\;\mathrm{for}\;\mathrm{all}\;v\in B_{c}^{\varphi }\right\},$$

where ${\left(L^{\Phi_{c}}\right)}^{*}$ denotes the dual space of $L^{\Phi_{c}}$. We know that, for all $u\in B_{c}^{\varphi }$ the normalizing function ψ is Gâteaux-differentiable and the Gâteaux-gradient is given by Equation (13). Hence, ∂ψ(u) consists of a single element and is given by

$$\partial \psi \left(u\right)=\frac{\varphi^{\prime }\left(c+u-\psi \left(u\right)u_{0}\right)}{\int_{T}u_{0}\varphi^{\prime }(c+u-\psi \left(u\right)u_{0}d\mu },\;u\in B_{c}^{\varphi }.$$

In fact, we prove below that Equation (13) belongs to ∂ψ(u), for all $u\in B_{c}^{\varphi }$.

**Proposition** **2.** *Let u be a function in domψ. Supposing that the functional*

(16) $$\frac{\varphi^{\prime }\left(c+u-\psi \left(u\right)u_{0}\right)}{\int_{T}u_{0}\varphi^{\prime }\left(c+u-\psi \left(u\right)u_{0}\right)d\mu }$$

*belongs to $L^{\Phi_{c}^{*}}$, then (16) belongs to ∂ψ(u).*

**Proof.**  We have that the functional (16) belongs to $L^{\Phi_{c}^{*}}$. Let *v* be a function in $B_{c}^{\varphi }$ such that $\int_{T}\varphi \left(c+u+v\right)d\mu <\infty$. In other words, u+v∈domψ, so we have that $\int_{T}\varphi \left(c+u-\psi \left(u\right)u_{0}\right)d\mu =1$ and $\int_{T}\varphi \left(c+u+v-\psi \left(u+v\right)u_{0}\right)d\mu =1$. Thus, by the convexity of φ, we have

$$\begin{array}{c} \int_{T}\left[v+\left(\psi \left(u+v\right)-\psi \left(u\right)\right)u_{0}\right]\varphi^{\prime }\left(c+u-\psi \left(u\right)u_{0}\right)d\mu \leq \;\\ \int_{T}\varphi \left(c+u-\psi \left(u\right)u_{0}\right)d\mu -\int_{T}\varphi \left(c+u+v-\psi \left(u+v\right)u_{0}\right)d\mu =0. \end{array}$$

Thus,

$$\int_{T}v\varphi^{\prime }\left(c+u-\psi \left(u\right)u_{0}\right)d\mu \leq \int_{T}u_{0}\varphi^{\prime }\left(c+u-\psi \left(u\right)u_{0}\right)d\mu \left(\psi \left(u+v\right)-\psi \left(u\right)\right)d\mu ,$$

and

(17) $$\frac{\int_{T}v\varphi^{\prime }\left(c+u-\psi \left(u\right)u_{0}\right)d\mu }{\int_{T}u_{0}\varphi^{\prime }\left(c+u-\psi \left(u\right)u_{0}\right)d\mu }\leq \psi \left(u+v\right)-\psi \left(u\right).$$

If $u+v\in B_{c}^{\varphi }\setminus \mathrm{dom}\;\psi$, then ψ(u+v)=∞, and

$$\frac{\int_{T}v\varphi^{\prime }\left(c+u-\psi \left(u\right)u_{0}\right)d\mu }{\int_{T}u_{0}\varphi^{\prime }\left(c+u-\psi \left(u\right)u_{0}\right)d\mu }<\psi \left(u+v\right)-\psi \left(u\right).$$

Consequently, Inequality (17) holds for all $v\in B_{c}^{\varphi }$ and the result follows. ☐

We need to find the subdifferential of ψ for *u* in the set ${\left\{\partial B_{c}^{\varphi }\right\}}_{<\infty }$. We know that ψ is a proper lower semicontinuous convex function, so

intdomψ⊂D(∂ψ)⊂domψ,

where $\mathrm{int}\;\mathrm{dom}\;\psi =B_{c}^{\varphi }$ and $D\left(\partial \psi \right)\setminus \mathrm{int}\;\mathrm{dom}\;\psi ={\left\{\partial B_{c}^{\varphi }\right\}}_{<\infty }$. As we have that intdomψ≠∅, then for u∈D(∂ψ)∖intdomψ, ∂ψ(u) is unbounded.

Since we are interested to prove that the set of functionals in Equation (13) is convex and these functionals are order continuous, we need to analyze only the order continuous part of the subdifferential, i.e., the part of the subdifferential that belongs to $L^{\Phi_{c}^{*}}$. We need to investigate whether the functional in Equation (16) belongs to $L^{\Phi_{c}^{*}}$, for $u\in {\left\{\partial B_{c}^{\varphi }\right\}}_{<\infty }$. For this, we will use some results.

**Lemma** **1** ([35], Lemma 3.11)**.** *Let $\Phi_{c}$ be a Musielak–Orlicz function that does not satisfy the $\Delta_{2}$-condition. In addition, assume that $\Phi_{c}\left(t,b_{\Phi }\left(t\right)\right)=\infty$ for μ-a.e. t∈T. Then there exist a strictly increasing sequence $0<\lambda_{n}↑1$, and sequences $\left\{u_{n}\right\}$ and $\left\{A_{n}\right\}$ of finite-valued, non-negative, measurable functions, and pairwise disjoint, measurable sets, respectively, such that*

$$I_{\Phi_{c}}\left(u_{n}\chi_{A_{n}}\right)=1,\;and\;I_{\Phi_{c}}\left(\lambda_{n}u_{n}\chi_{A_{n}}\right)\leq 2^{-n},\;for\;all\;n\geq 1.$$

**Proposition** **3** ([43], Proposition 2.3)**.** *Let* Φ *and* Ψ *be Musielak–Orlicz functions. Suppose that, for constants α, λ>0, there exists an integrable function h:T→[0,∞) such that*

αΨ(t,u)≤Φ(t,λu)+h(t),forallu≥0.

*Then, for constants $\alpha^{\prime }\in \left(0,\alpha \right)$ and $\lambda^{\prime }=\lambda$, or $\alpha^{\prime }=\alpha$ and $\lambda^{\prime }>\lambda$, a non-negative function $f\in {\tilde{L}}^{\Psi }$ can be found such that*

$$\alpha^{\prime }\Psi \left(t,u\right)\leq \Phi \left(t,\lambda^{\prime }u\right),\;\;\;for\;all\;u>f\left(t\right).$$

**Lemma** **2.** *Let $\Phi^{*}$ and $\Psi^{*}$ denote the complementary functions to the Musielak–Orlicz functions* Φ *and* Ψ*, respectively. Suppose that, for constants α,λ>0, there exists a non-negative function $f\in {\tilde{L}}^{\Psi }$ such that*

(18) αΨ(t,u)≤Φ(t,λu),forallu>f(t).

*Then, for constants $\alpha^{\prime }=1/\alpha$ and $\lambda^{\prime }>\lambda /\alpha$, or $\alpha^{\prime }\in \left(0,1/\alpha \right)$ and $\lambda^{\prime }=\lambda /\alpha$, a non-negative function $g\in {\tilde{L}}^{\Phi^{*}}$ can be found such that*

(19) $$\alpha^{\prime }\Phi^{*}\left(t,v\right)\leq \Psi^{*}\left(t,\lambda^{\prime }v\right),\;for\;all\;v>g\left(t\right).$$

**Proof.**  Defining the function h(t)=Ψ(t,f(t)), we can write

αΨ(t,u)≤Φ(t,λu)+αh(t),forallu≥0.

Calculating the Fenchel conjugate of the functions in the inequality above, we obtain

$$\frac{1}{\alpha }\Phi^{*}\left(t,v\right)\leq \Psi^{*}\left(t,\frac{\lambda }{\alpha }v\right)+h\left(t\right),\;\mathrm{for}\;\mathrm{all}\;v\geq 0.$$

From Proposition 3, we infer that Equation (19) is satisfied. ☐

**Lemma** **3.** *The $\Delta_{2}$-condition is equivalent to the statement that, for every λ∈(0,1), there exist a constant $\alpha_{\lambda }\in \left(0,1\right)$, and a non-negative function $f_{\lambda }\in {\tilde{L}}^{\Phi }$ such that*

(20) $$\alpha_{\lambda }\Phi \left(t,u\right)\leq \Phi \left(t,\lambda u\right),\;for\;all\;u>f_{\lambda }\left(t\right).$$

*The $\nabla_{2}$-condition is equivalent to the statement that, for any λ∈(0,1), there exist a constant $\gamma_{\lambda }>1$, and a non-negative function $f_{\lambda }\in {\tilde{L}}^{\Phi }$ such that*

(21) $$\gamma_{\lambda }\Phi \left(t,u\right)\leq \Phi \left(t,\lambda \gamma_{\lambda }u\right),\;for\;all\;u>f_{\lambda }\left(t\right).$$

**Proof.**  Suppose it satisfies the $\Delta_{2}$-condition. If the natural number n≥1 is such that $2^{-n}\leq \lambda$, then $\alpha^{n}\Phi \left(t,u\right)\leq \Phi \left(t,2^{-n}u\right)\leq \Phi \left(t,\lambda u\right)$, for all $u>2^{n-1}f\left(t\right)$. Conversely, if Φ satisfies Equation (20) and the natural number n≥1 is chosen so that $\lambda^{n}\leq 1/2$, then $\alpha_{\lambda }^{n}\Phi \left(t,u\right)\leq \Phi \left(t,\lambda^{n}u\right)\leq \Phi \left(t,\frac{1}{2}u\right)$, for all $u>\lambda^{-n+1}f_{\lambda }\left(t\right)$.

Assume that Equation (3) is satisfied. Let n≥1 be a natural number such that $2^{-n}\leq \lambda$. Then $\gamma^{n}\Phi \left(t,u\right)\leq \Phi \left(t,2^{-n}\gamma^{n}u\right)\leq \Phi \left(t,\lambda \gamma^{n}u\right)$, for all u>f(t). Conversely, if Equation (21) holds and the natural number n≥1 is chosen so that $\lambda^{n}\leq 1/2$, then $\gamma_{\lambda }^{n}\Phi \left(t,u\right)\leq \Phi \left(t,\lambda^{n}\gamma_{\lambda }^{n}u\right)\leq \Phi \left(t,\frac{1}{2}\gamma_{\lambda }^{n}u\right)$, for all u>f(t). ☐

The next result follows from Lemmas 2 and 3.

**Theorem** **1.** A Musielak–Orlicz function $\Phi_{c}$ satisfies the $\nabla_{2}$-condition if, and only if, its complementary function $\Phi^{*}$ satisfies the $\Delta_{2}$-condition.

**Proposition** **4.** Let $\Phi_{c}$ be a Musielak–Orlicz function that does not satisfy the $\Delta_{2}$-condition and that $\Phi (t,b_{\Phi_{c}}\left(t\right))=\infty$ for μ-a.e. t∈T. Then we can find a non-negative function $u\in {\tilde{L}}^{\Phi_{c}}$ such that $I_{\Phi_{c}^{*}}\left(\Phi_{c+}^{\prime }\left(t,u\left(t\right)\right)\right)=\infty$.

**Proof.**  Let $\left\{\lambda_{n}\right\}$, $\left\{u_{n}\right\}$ and $\left\{A_{n}\right\}$ be given as in Lemma 1. Select a subsequence $\left\{\lambda_{n_{k}}\right\}\subset \left\{\lambda_{n}\right\}$ for which the series $\sum_{k=1}^{\infty }\left(1-\lambda_{n_{k}}\right)$ converges, and $\left(1-\lambda_{n_{k}}\right)+2^{-n_{k}}<1$ for all k≥1. Because $\lambda \to I_{\Phi_{c}}\left(\lambda u_{n_{k}}\chi_{A_{n_{k}}}\right)$ is continuous for λ∈[0,1], we can find $\lambda_{k}^{\prime }\in \left(\lambda_{n_{k}},1\right)$ such that $I_{\Phi_{c}}\left(\lambda_{k}^{\prime }u_{n_{k}}\chi_{A_{n_{k}}}\right)=\left(1-\lambda_{n_{k}}\right)+2^{-n_{k}}$. Define $u=\sum_{k=1}^{\infty }\lambda_{k}^{\prime }u_{n_{k}}\chi_{A_{n_{k}}}$. Then, we can write

$$I_{\Phi_{c}}\left(u\right)=\sum_{k=1}^{\infty }I_{\Phi_{c}}\left(\lambda_{k}^{\prime }u_{n_{k}}\chi_{A_{n_{k}}}\right)=\sum_{k=1}^{\infty }\left[\left(1-\lambda_{n_{k}}\right)+2^{-n_{k}}\right]<\infty ,$$

and

$$\begin{array}{cc} \int_{T}u\left(t\right)\Phi_{c+}^{\prime }\left(t,u\left(t\right)\right)d\mu & =\sum_{k=1}^{\infty }\int_{A_{n_{k}}}\lambda_{k}^{\prime }u_{n_{k}}\left(t\right)\Phi_{c+}^{\prime }\left(t,\lambda_{k}^{\prime }u_{n_{k}}\left(t\right)\right)d\mu \\ & \geq \sum_{k=1}^{\infty }\lambda_{k}^{\prime }\frac{1}{\lambda_{k}^{\prime }-\lambda_{n_{k}}}\left[I_{\Phi_{c}}\left(\lambda_{k}^{\prime }u_{n_{k}}\chi_{A_{n_{k}}}\right)-I_{\Phi_{c}}\left(\lambda_{n_{k}}u_{n_{k}}\chi_{A_{n_{k}}}\right)\right]\\ & \geq \sum_{k=1}^{\infty }\frac{\lambda_{k}^{\prime }}{1-\lambda_{n_{k}}}\left[\left(1-\lambda_{n_{k}}\right)+2^{-n_{k}}-2^{-n_{k}}\right]\\ & =\sum_{k=1}^{\infty }\lambda_{k}^{\prime }=\infty . \end{array}$$

Hence, it follows that

$$I_{\Phi_{c}^{*}}\left(\Phi_{c+}^{\prime }\left(t,u\left(t\right)\right)\right)=\int_{T}u\left(t\right)\Phi_{c+}^{\prime }\left(t,u\left(t\right)\right)d\mu -I_{\Phi_{c}}\left(u\right)=\infty ,$$

which concludes the proof. ☐

The previous proposition makes it clear that we can find a $u\in {\tilde{L}}^{\Phi_{c}}$, but $\Phi_{c+}^{\prime }\left(t,u\left(t\right)\right)\notin {\tilde{L}}^{\Phi_{c}^{*}}$. Let *u* be as in Proposition 4, clearly for λ∈(0,1), $I_{\Phi_{c}}\left(\lambda u\right)<\infty$ and for λ>1, $I_{\Phi_{c}}\left(\lambda u\right)=\infty$ ([35], Remark 3.12).

**Proposition** **5.** *Let $\Phi_{c}$ be a Musielak–Orlicz function such that, $\Phi_{c}$ satisfies $\nabla_{2}$-condition, does not satisfy $\Delta_{2}$-condition and $\Phi_{c}\left(t,b_{\Phi }\left(t\right)\right)=\infty$. Then we can find $w\in \partial B_{c}^{\varphi }$ such that*

(22) $$\frac{\varphi^{\prime }\left(c+w-\psi \left(w\right)u_{0}\right)}{\int_{T}u_{0}\varphi^{\prime }\left(c+w-\psi \left(w\right)u_{0}\right)d\mu }\notin L^{\Phi_{c}^{*}}$$

*where ${\tilde{L}}^{\Phi_{c}^{*}}$ is the Musielak–Orlicz class of $\Phi_{c}^{*}$, the conjugate of $\Phi_{c}$.*

**Proof.**  Take $k_{0}\geq 1$ and denote $B=T\setminus {⋃}_{k=k_{0}}^{\infty }A_{n_{k}}$, then we define $\tilde{u}=\sum_{k=k_{0}}^{\infty }\lambda_{k}^{\prime }u_{n_{k}}\chi_{A_{n_{k}}}$. We can choose $\lambda^{\prime }<0$ such that

$$w=\lambda^{\prime }u_{0}\chi_{B}+\tilde{u}$$

satisfies $\int_{T}w\varphi^{\prime }\left(c\right)d\mu =0$. In other words, $w\in B_{c}^{\varphi }$. It is easy to see that $\int_{T}\varphi \left(c+\alpha w\right)d\mu <\infty$ for α∈(0,1) and $\int_{T}\varphi \left(c+\alpha w\right)d\mu =\infty$ for α>1, so $w\in \partial B_{c}^{\varphi }$. The need to show Equation (22) remains. From Proposition 4 we have that

$$\begin{array}{cc} \int_{T}w\Phi_{c+}^{\prime }\left(t,w\left(t\right)\right)d\mu = & \int_{B}\lambda^{\prime }u_{0}\left(t\right)\Phi_{c+}^{\prime }\left(t,\lambda^{\prime }u_{0}\right)d\mu +\sum_{k=k_{0}}^{\infty }\int_{A_{n_{k}}}I_{\Phi_{c}}\left(\lambda_{k}^{\prime }u_{n_{k}}\chi_{A_{n_{k}}}\right)d\mu =\infty , \end{array}$$

since

$$\begin{array}{cc} \int_{B}\lambda^{\prime }u_{0}\left(t\right)\Phi_{c+}^{\prime }\left(t,\lambda^{\prime }u_{0}\right)d\mu & \leq \lambda^{\prime }\int_{B}\Phi_{c}\left(\left(\lambda^{\prime }+1\right)u_{0}\right)-\Phi_{c}\left(\lambda^{\prime }u_{0}\right)d\mu \\ & \leq \lambda^{\prime }\int_{B}\varphi \left(c+\left(\lambda^{\prime }+1\right)u_{0}\right)-\varphi \left(c+\lambda^{\prime }u_{0}\right)d\mu \\ & <\infty . \end{array}$$

Thus,

$$I_{\Phi_{c}^{*}}\left(\Phi_{c+}^{\prime }\left(t,w\left(t\right)\right)\right)=\int_{T}w\left(t\right)\Phi_{c+}^{\prime }\left(t,w\left(t\right)\right)d\mu -I_{\Phi_{c}}\left(w\right)=\infty ,$$

consequently $\varphi^{\prime }\left(c+w\right)\notin {\tilde{L}}^{\Phi_{c}^{*}}$. Since $\Phi_{c}\in \nabla_{2}$, we have that $\Phi_{c}^{*}\in \Delta_{2}$ and therefore ${\tilde{L}}^{\Phi_{c}^{*}}=L^{\Phi_{c}^{*}}$. We conclude that $\varphi^{\prime }\left(c+w\right)\notin L^{\Phi_{c}^{*}}$. Since $L^{\Phi_{c}^{*}}$ is a linear set, we have that Equation (22) occurs. ☐

As a consequence of Proposition 5, we have that it is possible to find $u\in {\left\{\partial B_{c}^{\varphi }\right\}}_{<\infty }$ such that

$$\frac{\varphi^{\prime }\left(c+u-\psi \left(u\right)u_{0}\right)}{\int_{T}u_{0}\varphi^{\prime }\left(c+u-\psi \left(u\right)u_{0}\right)d\mu }\notin L^{\Phi_{c}^{*}},$$

and therefore the functional in Equation (16) does not belong to ∂ψ(u).

We conclude in this section that, if the functional

(23) $$\frac{\varphi^{\prime }\left(c+u-\psi \left(u\right)u_{0}\right)}{\int_{T}u_{0}\varphi^{\prime }\left(c+u-\psi \left(u\right)u_{0}\right)d\mu }$$

belongs to $L^{\Phi_{c}^{*}}$, then the functional belongs to ∂ψ(u) for u∈domψ.

In next section we finally prove that the set of functionals formed by Gâteaux gradient of the normalizing function ψ that belongs to $L^{\Phi_{c}^{*}}$ is convex, so we can guarantee that the generalized mixture arc is well defined.

### 3.2. Convexity of the Functionals Set

We already know that, for the generalized mixture arc in Equation (11) to be well defined, it is necessary that the set of functionals

(24) $$\left\{\frac{\varphi^{\prime }\left(c+u-\psi \left(u\right)u_{0}\right)}{\int_{T}u_{0}\varphi^{\prime }\left(c+u-\psi \left(u\right)u_{0}\right)d\mu },\;\;\;u\in \mathrm{dom}\;\psi \right\}⋂L^{\Phi_{c}^{*}}$$

to be convex. From Proposition 2, the set in Equation (24) is contained in the range of ∂ψ, the set given by

(25) $$\mathrm{range}\;\partial \psi =⋃\left\{\partial \psi (u):u\in \mathrm{dom}\;\psi \right\}.$$

Let $\psi^{*}$ be the conjugate function of ψ. By the fact that $\psi^{*}$ be a l.s.c. proper convex function, $\mathrm{int}\;\mathrm{dom}\;\psi^{*}$ and $\mathrm{dom}\;\psi^{*}$ are convex sets and the range of ∂ψ is the effective domain of $\partial \psi^{*}$, since ${\left(\partial \psi \right)}^{-1}=\partial \psi^{*}$. Thus

(26) $$\mathrm{int}\;\mathrm{dom}\;\psi^{*}\subset D\left(\partial \psi^{*}\right)\subset \mathrm{dom}\;\psi^{*}$$

is the same that

(27) $$\mathrm{int}\;\mathrm{dom}\;\psi^{*}\subset \mathrm{range}\;\partial \psi \subset \mathrm{dom}\;\psi^{*}.$$

To prove that the set in Equation (24) is convex, we analyze the set in Equation (25) in three cases. Let $u^{*},v^{*}$ be elements in Equation (25) such that

Case 1. $u^{*},v^{*}\in \mathrm{int}\;\mathrm{dom}\;\psi^{*}$, so by convexity of $\mathrm{int}\;\mathrm{dom}\;\psi^{*}$, for λ∈(0,1), we have $\lambda u^{*}+\left(1-\lambda \right)v^{*}\in \mathrm{int}\;\mathrm{dom}\;\psi^{*}$.
Case 2. If $u^{*}\in \mathrm{int}\;\mathrm{dom}\;\psi^{*}$ and $v^{*}\in D\left(\partial \psi^{*}\right)\setminus \mathrm{int}\;\mathrm{dom}\;\psi^{*}$, then $\lambda u^{*}+\left(1-\lambda \right)v^{*}\in \mathrm{int}\;\mathrm{dom}\;\psi^{*}$, for λ∈(0,1) ([41], Fact 2.1).
Case 3. Let $u^{*},v^{*}$ be elements in Equation (25) belonging to $D\left(\partial \psi^{*}\right)\setminus \mathrm{int}\;\mathrm{dom}\;\psi^{*}$.

We want to prove that, for λ∈(0,1), $\lambda u^{*}+\left(1-\lambda \right)v^{*}$ belongs to Equation (25). To solve this problem, we are going to prove that $D\left(\partial \psi^{*}\right)=\mathrm{int}\;\mathrm{dom}\;\psi^{*}$. Supposing φ a strictly convex function, then ψ is a strictly convex function. In next proposition, we show that $\partial \psi^{*}\left(u^{*}\right)$ is a unitary set.

**Proposition** **6.** Let ψ be a strictly convex function, then $\partial \psi^{*}\left(u^{*}\right)$ is a unitary set, where $u^{*}\in \partial \psi \left(u\right)$, with u∈D(∂ψ).

**Proof.**  Assuming that ψ is a strictly convex function we have that for λ∈(0,1) and $\forall u_{1}\neq u_{2}\in \mathrm{dom}\;\psi$

(28) $$\psi \left(\lambda u_{1}+\left(1-\lambda \right)u_{2}\right)<\lambda \psi \left(u_{1}\right)+\left(1-\lambda \right)\psi \left(u_{2}\right).$$

Supposing that $\partial \psi^{*}\left(u^{*}\right)$ is not a unitary set, i.e., $\partial \psi^{*}\left(u^{*}\right)=\left\{u_{1},u_{2},\ldots \right\}$, where $u_{i}^{*}\in D\left(\partial \psi \right)$, i=1,2,…. Taking $u_{1}$, $u_{2}\in \partial \psi^{*}\left(u^{*}\right)$. By Young’s Inequality (10)

(29) $$\left(\lambda u_{1}+\left(1-\lambda \right)u_{2},u^{*}\right)\leq \psi \left(\lambda u_{1}+\left(1-\lambda \right)u_{2}\right)+\psi^{*}\left(u^{*}\right),$$

where λ∈(0,1) and as a consequence of $u_{1},u_{2}\in \partial \psi^{*}\left(u^{*}\right)$ we have

(30) $$\begin{array}{cc} \psi \left(u_{1}\right)+\psi^{*}\left(u^{*}\right) & =(u_{1},u^{*}), \end{array}$$

and

(31) $$\begin{array}{cc} \psi \left(u_{2}\right)+\psi^{*}\left(u^{*}\right) & =(u_{1},u^{*}). \end{array}$$

Taking the product of Equation (30) by λ, the product of Equation (31) by (1−λ) and adding the two obtained equations, we have

(32) $$\begin{array}{cc} \lambda \psi \left(u_{1}\right)+\left(1-\lambda \right)\psi \left(u_{2}\right)+\psi^{*}\left(u^{*}\right) & =(\lambda u_{1}+\left(1-\lambda \right)u_{2},u^{*}). \end{array}$$

From Equations (29) and (32), we obtain

(33) $$\lambda \psi \left(u_{1}\right)+\left(1-\lambda \right)\psi \left(u_{2}\right)\leq \psi \left(\lambda u_{1}+\left(1-\lambda \right)u_{2}\right),$$

which is a contradiction by Equation (28). This implies that $\partial \psi^{*}\left(u^{*}\right)$ is a unitary set and this completes the proof. ☐

Thus, the set $\partial \psi^{*}\left(u^{*}\right)$ is unitary, then $\partial \psi^{*}$ is locally bounded at $u^{*}\in D\left(\partial \psi^{*}\right)$ and, therefore, by Fact 1, we conclude that $u^{*}\in \mathrm{int}\;\mathrm{dom}\;\psi^{*}$ which implies that $D\left(\partial \psi^{*}\right)\subset \mathrm{int}\;\mathrm{dom}\;\psi^{*}$, by Equation (26), we have that

(34) $$\mathrm{range}\;\partial \psi =D\left(\partial \psi^{*}\right)=\mathrm{int}\;\mathrm{dom}\;\psi^{*}.$$

Therefore, by Fact 2, there exists no functional $u^{*}$ in Equation (25) such that $u^{*}\in D\left(\partial \psi^{*}\right)\setminus \mathrm{int}\;\mathrm{dom}\;\psi^{*}$. Thus Equation (25) is a convex set and, as a consequence, the generalized mixture arc is well defined, since the set in Equation (24) is a convex set. Indeed, let *u*, *v* be functions in domψ such that

(35) $$u^{*}=\frac{\varphi^{\prime }\left(c+u-\psi \left(u\right)u_{0}\right)}{\int_{T}u_{0}\varphi^{\prime }\left(c+u-\psi \left(u\right)u_{0}\right)d\mu }$$

and

(36) $$v^{*}=\frac{\varphi^{\prime }\left(c+v-\psi \left(v\right)u_{0}\right)}{\int_{T}u_{0}\varphi^{\prime }\left(c+v-\psi \left(v\right)u_{0}\right)d\mu }$$

belong to Equation (24). Clearly,

$$\int_{T}u_{0}u^{*}d\mu =\frac{\int_{T}u_{0}\varphi^{\prime }\left(c+u-\psi \left(u\right)u_{0}\right)d\mu }{\int_{T}u_{0}\varphi^{\prime }\left(c+u-\psi \left(u\right)u_{0}\right)d\mu }=1$$

and

$$\int_{T}u_{0}v^{*}d\mu =\frac{\int_{T}u_{0}\varphi^{\prime }\left(c+v-\psi \left(v\right)u_{0}\right)d\mu }{\int_{T}u_{0}\varphi^{\prime }\left(c+v-\psi \left(v\right)u_{0}\right)d\mu }=1.$$

We note that, the functionals in Equation (24) are the only elements in Equation (25) that satisfy $\int_{T}u_{0}u^{*}d\mu =1$. For λ∈(0,1) we have

$$\int_{T}u_{0}\left(\left(1-\lambda \right)u^{*}+\lambda v^{*}\right)d\mu =1,$$

then there exist functions $w_{\lambda }\in \mathrm{dom}\;\psi$ such that

$$\frac{\varphi^{\prime }\left(c+w_{\lambda }-\psi \left(w_{\lambda }\right)u_{0}\right)d\mu }{\int_{T}u_{0}\varphi^{\prime }\left(c+w_{\lambda }-\psi \left(w_{\lambda }\right)u_{0}\right)d\mu }=\left(1-\lambda \right)u^{*}+\lambda v^{*},\;\mathrm{for}\;\mathrm{each}\;\lambda \in \left(0,1\right).$$

Thus, the set in Equation (24) is a convex set.

In this section, we proved that the generalized mixture arc is well defined for a deformed exponential φ strictly convex. In the next section, we discuss generalized open exponential arcs and generalized open mixture arcs.

## 4. Generalized Arcs

The concept of arc-connected probability distributions was defined by de Souza et al. [36] defined the concept of arc-connected probability distributions. Fixing any deformed exponential φ we say that two probability distributions $p,q\in P_{\mu }$ are φ-connected if, for each α∈[0,1], there exists k(α):=k(α;p,q)∈R such that

$$\int_{T}\varphi \left(\alpha \varphi^{-1}\left(p\right)+\left(1-\alpha \right)+k\left(\alpha \right)u_{0}\right)d\mu =1.$$

In [31], necessary and sufficient conditions for any probability distributions being φ-connected were provided. In this section, we discuss the concept of two probability distributions $p,q\in P_{\mu }$ are φ-connected by open arcs. We generalize open exponential arcs and open mixture arcs, defined in [22] and studied later in [23].

### 4.1. Generalized Open Exponential Arcs

Let us define the generalized open arcs and prove some of its properties.

**Definition** **1.** *For a fixed deformed exponential φ, we say that p and q in $P_{\mu }$ are φ-connected by an open arc if there exists an open interval I⊃[0,1] and a constant k(α) such that*

$$p\left(\alpha \right)=\varphi (\left(1-\alpha \right)\varphi^{-1}\left(p\right)+\alpha \varphi^{-1}\left(q\right)+k\left(\alpha \right)u_{0})$$

*belongs to $P_{\mu }$ for every t∈I, where k(α) depends of t,pandq.*

In the following proposition, we give an equivalent definition of φ-connection by open arc.

**Proposition** **7.** $p,q\in P_{\mu }$ are φ-connected by an open arc if and only if there exist an open interval I⊃[0,1] and a random variable $v\in L_{c}^{\varphi }$, such that p(α)∝φ(c+αv) belongs to $P_{\mu }$, for all t∈I and p(0)=p and p(1)=q.

**Proof.**  Let us assume that p,q are φ-connected, i.e., $\int_{T}\varphi \left(\left(1-\alpha \right)\varphi^{-1}\left(p\right)+\alpha \varphi^{-1}\left(q\right)\right)d\mu <\infty$, for all α∈I. Since

$$\begin{array}{cc} \int_{T}\varphi \left(\left(1-\alpha \right)\varphi^{-1}\left(p\right)+\alpha \varphi^{-1}\left(q\right)\right)d\mu & =\int_{T}\varphi \left(\alpha \left[\varphi^{-1}\left(q\right)-\varphi^{-1}\left(p\right)\right]+\varphi^{-1}\left(p\right)\right)d\mu \\ & =\int_{T}\varphi \left(c+\alpha v\right)d\mu , \end{array}$$

where $v=\varphi^{-1}\left(q\right)-\varphi^{-1}\left(p\right)$ and φ(c)=p, then $v\in L_{c}^{\varphi }$. Moreover, p(α)∝φ(c+αv) belongs to $P_{\mu }$, for every α∈I and p(0)=φ(c)=p and p(1)=q. The converse follows immediately. Suppose that q=p(1), we have φ(c+v)=q, then $v=\varphi^{-1}\left(q\right)-\varphi^{-1}\left(p\right)$, with φ(c)=p=p(0). ☐

Because of $v\in L_{c}^{\varphi }$ the need to define the open arcs arises. As a consequence of Proposition 7, we have that if $p,q\in P_{\mu }$ are φ- connected by an open arc, then the random variable $v\in K_{c}^{\varphi }$, since $\int_{T}\varphi \left(c+\alpha v\right)d\mu <\infty$ for all α∈(−ε,1+ε). With this, we can prove the following results.

**Corollary** **1.** Let $p,q\in P_{\mu }$, where p=φ(c). We have that $q\in F_{c}^{\varphi }$ if and only if, p and q are φ-connected by an open arc.

**Proof.**  Supposing $q\in F_{c}^{\varphi }$, then $q=\varphi (c+v-\psi \left(v\right)u_{0})$ where $v\in B_{c}^{\varphi }$. Thus, we have $\int_{T}\varphi \left(c+\alpha v\right)d\mu <\infty$ for all α∈(−ε,1+ε), we deduce that p(α)∝φ(c+αv) is an open arc containing *p* and *q*. Conversely, supposing that *p* and *q* are φ-connected by an open arc, by Proposition 7, there exist an open interval I⊃[0,1] and $v\in K_{c}^{\varphi }$ such that p(α)∝φ(c+αv) belongs to $P_{\mu }$ with q=p(1). If $v\in B_{c}^{\varphi }$, then $q=\varphi \left(c+v\right)\in F_{c}^{\varphi }$ and the proof is over. Otherwise, let *w* be such that

$$w=v-\frac{\int_{T}v\varphi^{\prime }\left(c\right)d\mu }{\int_{T}u_{0}\varphi^{\prime }\left(c\right)d\mu }u_{0},$$

thus $\int_{T}w\varphi^{\prime }\left(c\right)d\mu =0$ and $w\in B_{c}^{\varphi }$. Hence, we have q=φ(c+v)=φ(c+w) and $q\in F_{c}^{\varphi }$. ☐

With this, we prove that, for φ(c)=p, the φ-family of probability distributions $F_{c}^{\varphi }$ is the set of all $q\in P_{\mu }$ such that *q* is φ-connected by an open arc to *p*.

**Corollary** **2.** Let p=φ(c) and $q=\varphi (\tilde{c})$ be such that $p,q\in P_{\mu }$ are φ -connected by an open arc. Then the spaces $L_{c}^{\varphi }$ and $L_{\tilde{c}}^{\varphi }$ are equal as sets.

**Proof.**  It follows from Corollary 1 that *p* and *q* are in the same φ-family, then $\tilde{c}=c+u-\psi \left(u\right)u_{0}$ and by Vigelis and Cavalcante [28], Lemma 5, it follows the result. ☐

Now, we show that the connection by generalized open exponential arcs is an equivalence relation.

**Proposition** **8.** The relation in Definition 1 is an equivalence relation.

**Proof.**  Reflexive and symmetry properties follow from the definition and now, we prove transitivity. Consider $p,q,r\in P_{\mu }$

p(t)∝φ(c+tu),r(t)∝φ(c+tv),t∈(−ε,1+ε),

with p(0)=φ(c)=p, p(1)=φ(c+u)=q, r(0)=φ(c)=p, r(1)=φ(c+v)=r with $u,v\in L_{c}^{\varphi }$. We have that *p* is φ-connected to *q* and *r*, respectively. We need to prove that *q* and *r* are also φ-connected. Consider

q(t)∝φ(c+(1−t)u+tv)∝φ(c+u+t(v−u))

is defined with $c+u=\tilde{c}$, $p\left(t\right)\propto \varphi (\tilde{c}+t\left(v-u\right))$, $v-u\in L_{c}^{\varphi }$, such that $q\left(0\right)=\varphi \left(\tilde{c}\right)=\varphi \left(c+u\right)=q$, $q\left(1\right)=\varphi (\tilde{c}+\left(v-u\right))=\varphi \left(c+v\right)=r$. Therefore, *q* and *r* are φ-connected. ☐

We know from Corollary 1 that the φ-family $F_{c}^{\varphi }$ coincides with the set of all $q\in P_{\mu }$ which are φ-connected to *p* by an open arc. We want now to prove that the φ-family $F_{c}^{\varphi }$ is convex for some deformed exponential φ.

**Lemma** **4.** *Let φ be a fixed deformed exponential. Assuming that ${\left(\varphi^{-1}\right)}^{\prime \prime }\left(x\right)$ is continuous and*

(37) $$\frac{\alpha \varphi^{\prime \prime }\left(\alpha \varphi^{-1}\left(x\right)+k\right)}{\varphi^{\prime \prime }\left(\alpha \varphi^{-1}\left(x\right)+k\right)}\geq \frac{\varphi^{\prime \prime }\left(\varphi^{-1}\left(x\right)\right)}{\varphi^{\prime \prime }\left(\varphi^{-1}\left(x\right)\right)},$$

*then $F\left(x\right)=\varphi (\alpha \varphi^{-1}\left(x\right)+k)$, for some fixed α>1 and k∈R is a convex function.*

**Proof.**  We know that, if $F^{\prime \prime }\left(x\right)\geq 0$∀α>1 and ∀x, then F(x) is a convex function. We have

$$F^{\prime \prime }\left(x\right)=\frac{\alpha^{2}\varphi^{\prime \prime }\left(\alpha \varphi^{-1}\left(x\right)+k\right)\varphi^{\prime }\left(\varphi^{-1}\left(x\right)\right)-\alpha \varphi^{\prime }\left(\alpha \varphi^{-1}\left(x\right)+k\right)\varphi^{\prime \prime }\left(\varphi^{-1}\left(x\right)\right)}{{\left[\varphi^{\prime }\left(\alpha \varphi^{-1}\left(x\right)\right)\right]}^{3}}$$

by the fact that φ is an increasing function ${\left[\varphi^{\prime }\left(\alpha \varphi^{-1}\left(x\right)\right)\right]}^{3}>0$. Hence, we have $F^{\prime \prime }\left(x\right)\geq 0$ if and only if

$$\alpha^{2}\varphi^{\prime \prime }\left(\alpha \varphi^{-1}\left(x\right)+k\right)\varphi^{\prime }\left(\varphi^{-1}\left(x\right)\right)-\alpha \varphi^{\prime }\left(\alpha \varphi^{-1}\left(x\right)+k\right)\varphi^{\prime \prime }\left(\varphi^{-1}\left(x\right)\right)\geq 0,$$

which follows from Equation (37). ☐

**Proposition** **9.** *Let $p\in P_{\mu }$ such that φ(c)=p. Assuming that ${\left(\varphi^{-1}\right)}^{\prime \prime }\left(x\right)$ is continuous and*

(38) $$\frac{\alpha \varphi^{\prime \prime }\left(\alpha \varphi^{-1}\left(x\right)+k\right)}{\varphi^{\prime \prime }\left(\alpha \varphi^{-1}\left(x\right)+k\right)}\geq \frac{\varphi^{\prime \prime }\left(\varphi^{-1}\left(x\right)\right)}{\varphi^{\prime \prime }\left(\varphi^{-1}\left(x\right)\right)}$$

*for some fixed α>1 and k∈R. Then, the *φ*-family of probability $F_{c}^{\varphi }$ is convex.*

**Proof.**  Note that, for any $\varphi \left(\tilde{c}\right)=r\in F_{c}^{\varphi }$, $F_{c}^{\varphi }=F_{\tilde{c}}^{\varphi }$. Suppose $q\in F_{c}^{\varphi }$, and consider p(λ)=λp+(1−λ)q for any λ∈[0,1]. We show that $p\left(\lambda \right)\in F_{c}^{\varphi },\;\forall \lambda \in \left[0,1\right]$ by proving that $\int_{T}\varphi (\left(1-\alpha \right)\varphi^{-1}\left(p\right)+\alpha \varphi^{-1}\left(p\left(\lambda \right)\right)d\mu <\infty$ for α∈(−ε,1+ε). In the others words, we will show that p(λ) and *p* are φ-connected for all λ∈[0,1].

For α∈(0,1), due the convexity of φ, we have

$$\begin{array}{cc} \int_{T}\varphi \left(\left(1-\alpha \right)\varphi^{-1}\left(p\right)+\alpha \varphi^{-1}\left(p\left(\lambda \right)\right)\right)d\mu & \leq \int_{T}\left(1-\alpha \right)\varphi \left(\varphi^{-1}\left(p\right)\right)+\alpha \varphi \left(\varphi^{-1}\left(p\left(\lambda \right)\right)\right)d\mu \\ & =\int_{T}\left(1-\alpha \right)p+\alpha p\left(\lambda \right)d\mu \\ & =\left(1-\alpha \right)\int_{T}pd\mu +\alpha \int_{T}p\left(\lambda \right)d\mu \\ & =1. \end{array}$$

If α∈(−ε,0), according the convexity of $\alpha \varphi^{-1}\left(x\right)$ and φ(x), we have

$$\begin{array}{c} \int_{T}\varphi \left(\left(1-\alpha \right)\varphi^{-1}\left(p\right)+\alpha \varphi^{-1}\left(p\left(\lambda \right)\right)\right)d\mu \;\\ \begin{array}{c} \leq \int_{T}\varphi \left(\lambda \alpha \varphi^{-1}\left(p\right)+\left(1-\lambda \right)\alpha \varphi^{-1}\left(q\right)+\left(1-\alpha \right)\varphi^{-1}\left(p\right)\right)d\mu \\ =\int_{T}\varphi \left(\lambda \left[\alpha \varphi^{-1}\left(p\right)+\left(1-\alpha \right)\varphi^{-1}\left(p\right)\right]+\left(1-\lambda \right)\left[\alpha \varphi^{-1}\left(q\right)+\left(1-\alpha \right)\varphi^{-1}\left(p\right)\right]\right)d\mu \\ \leq \int_{T}\lambda \varphi \left(\alpha \varphi^{-1}\left(p\right)+\left(1-\alpha \right)\varphi^{-1}\left(p\right)\right)+\left(1-\lambda \right)\varphi \left(\alpha \varphi^{-1}\left(q\right)+\left(1-\alpha \right)\varphi^{-1}\left(p\right)\right)d\mu \\ =\lambda \int_{T}\varphi \left(\varphi^{-1}\left(p\right)\right)d\mu +\left(1-\lambda \right)\int_{T}\varphi \left(\alpha \varphi^{-1}\left(q\right)+\left(1-\alpha \right)\varphi^{-1}\left(p\right)\right)d\mu \\ =\lambda +\left(1-\lambda \right)\int_{T}\varphi \left(\alpha \varphi^{-1}\left(q\right)+\left(1-\alpha \right)\varphi^{-1}\left(p\right)\right)d\mu , \end{array} \end{array}$$

since $q\in F_{c}^{\varphi }$, we have by Corollary 1 that *q* and *p* are φ-connected. Hence,

$$\int_{T}\varphi \left(\left(1-\alpha \right)\varphi^{-1}\left(p\right)+\alpha \varphi^{-1}\left(p\left(\lambda \right)\right)\right)d\mu <\infty$$

so p(λ) and *p* are φ-connected by an open arc, for all α∈(−ε,0).

Now, if α∈(1,1+ε), the Lemma 4, $F\left(x\right)=\varphi (\alpha \varphi^{-1}\left(x\right)+k)$ is a convex function, so

(39) $$\varphi \left(\alpha \varphi^{-1}\left(\lambda x+\left(1-\lambda \right)y\right)+k\right)\leq \lambda \varphi \left(\alpha \varphi^{-1}\left(x\right)+k\right)+\left(1-\lambda \right)\varphi \left(\alpha \varphi^{-1}\left(y\right)+k\right),$$

where λ∈[0,1] and *k* a constant. Taking $k=\left(1-\alpha \right)\varphi^{-1}\left(p\right)$, we have

$$\begin{array}{cc} \int_{T}\varphi \left(\alpha \varphi^{-1}\left(p\left(\lambda \right)\right)+\left(1-\alpha \right)\varphi^{-1}\left(p\right)\right)d\mu & \leq \lambda \int_{T}\varphi (\alpha \varphi^{-1}\left(p\right)+\left(1-\alpha \right)\varphi^{-1}\left(p\right)d\mu \\ & \;+\left(1-\lambda \right)\int_{T}\varphi (\alpha \varphi^{-1}\left(q\right)+\left(1-\alpha \right)\varphi^{-1}\left(p\right)d\mu \\ & =\lambda +\left(1-\lambda \right)\int_{T}\varphi \left(\alpha \varphi^{-1}\left(q\right)+\left(1-\alpha \right)\varphi^{-1}\left(p\right)\right)d\mu \\ & <\infty , \end{array}$$

since $q\in F_{c}^{\varphi }$ and, therefore, *p* and *q* are φ-connected by an open arc. ☐

### 4.2. Generalized Open Mixture Arcs

In Section 3.2, we proved that the generalized mixture arc given by

(40) $$p\left(\alpha \right)=F^{-1}\left((1-\alpha )F(p)+\alpha F(q)\right),$$

is well defined for α∈[0,1]. In this section, our goal is twofold: firstly, to ensure that the open arc is also well defined; and, secondly, to provide some properties of these arcs. For such objectives, we use Equation (34), which establishes that $D(\partial \psi^{*})=\mathrm{range}\;\partial \psi$ is an open set, so we can extend the convex combination in Equation (40) between F(p) and F(q) beyond these extreme points while maintaining positivity of (1−α)F(p)+αF(q). Indeed, by the fact $D(\partial \psi^{*})=\mathrm{range}\;\partial \psi$ is an open set, so there exists $\varepsilon_{1}>0$ such that $B\left(F\left(p\right),\varepsilon_{1}\right)$ is the open ball of radius $\varepsilon_{1}$ centered at F(p) with $B\left(F\left(p\right),\varepsilon_{1}\right)\subset D\left(\partial \psi^{*}\right)$. Similarly, there exists $\varepsilon_{2}>0$ such that $B\left(F\left(q\right),\varepsilon_{2}\right)\subset D\left(\partial \psi^{*}\right)$. Taking $\varepsilon =\min\{\varepsilon_{1},\varepsilon_{2}\}$ we guarantee that the combination (1−α)F(p)+αF(q) in (40) can be extended to α∈I=(−ε,1+ε)⊃[0,1].

**Definition** **2.** *For a fixed deformed exponential φ, we say that p and q in $P_{\mu }$ are φ-connected by an open mixture arc if there exists an open interval I⊃[0,1] such that*

(41) $$p\left(\alpha \right)=F^{-1}\left((1-\alpha )F(p)+\alpha F(q)\right)$$

*belongs to $P_{\mu }$ for every α∈I, where $F\left(p\right)=\frac{\varphi^{\prime }\left(\varphi^{-1}\left(p\right)\right)}{\int_{T}u_{0}\varphi^{\prime }\left(\varphi^{-1}\left(p\right)\right)d\mu }$.*

In [22], it was shown that densities connected by open mixture arcs have bounded away from zero ratios. Santacroce et al. [23] showed the converse implication, providing a characterization of open mixture models. Here, one can see that the fundamental role for being connected by open mixture arcs is given by ratios $\frac{F(p)}{F(q)}$ which have to be bounded. The functional F(p) in the definition of generalized open mixture arc satisfies F(p)>0. Thus the combination (1−α)F(p)+αF(q) in (41) has to satisfy the same property, that is, (1−α)F(p)+αF(q)>0. Assume that *p* and *q* are φ-connected by an open mixture arc given according to (41) belong to $P_{\mu }$ for all $\alpha \in \left(-\varepsilon_{1},1+\varepsilon_{2}\right)⊃\left[-\varepsilon ,1+\varepsilon \right]$ with ε>0. Since p(−ε) and $p\left(1+\varepsilon \right)\in P_{\mu }$, then

F(p(−ε))=(1+ε)F(p)+(−ε)F(q)>0,

which implies that

(42) $$\frac{F(p)}{F(q)}>\frac{\varepsilon }{1+\varepsilon }$$

and

F(p(1+ε))=(−ε)F(p)+(1+ε)F(q)>0,

which give to us

(43) $$\frac{F(p)}{F(q)}<\frac{1+\varepsilon }{\varepsilon }.$$

Combining inequalities (42) and (43),we have

(44) $$\frac{\varepsilon }{1+\varepsilon }<\frac{F(p)}{F(q)}<\frac{1+\varepsilon }{\varepsilon }.$$

Conversely, if we have Equation (44), then (1−α)F(p)+αF(q)>0 and Equation (41) belongs to $P_{\mu }$. Thus, we have that *p* and *q* in $P_{\mu }$ are φ-connected by an open mixture arc if and only if the ratio $\frac{F(p)}{F(q)}$ is bounded. By the fact that range∂ψ is an open set, there exists an interval I⊃[0,1] such that (1−α)F(p)+αF(q) belongs to range∂ψ and we have

$$\int_{T}u_{0}\left[(1-\alpha )F(p)+\alpha F(q)\right]d\mu =1,\;\mathrm{for}\;\mathrm{all}\;\alpha \in I⊃\left[0,1\right],$$

for all α∈I. Then, there exist functions $w_{\alpha }\in \mathrm{dom}\;\psi$ such that

$$\left(1-\alpha \right)F\left(p\right)+\alpha F\left(q\right)=\frac{\varphi^{\prime }\left(c+w_{\alpha }-\psi \left(w_{\alpha }\right)u_{0}\right)}{\int_{T}u_{0}\varphi^{\prime }\left(c+w_{\alpha }-\psi \left(w_{\alpha }\right)u_{0}\right)d\mu },$$

with

$$p\left(\alpha \right)=F^{-1}\left(\frac{\varphi^{\prime }\left(c+w_{\alpha }-\psi \left(w_{\alpha }\right)u_{0}\right)}{\int_{T}u_{0}\varphi^{\prime }\left(c+w_{\alpha }-\psi \left(w_{\alpha }\right)u_{0}\right)d\mu }\right),\;\mathrm{for}\;\mathrm{all}\;\alpha \in I⊃\left[0,1\right],$$

that is, the convex combination in Equation (41) is also a functional of the type in Equation (12) for all α∈I. Then, the open mixture arc is well-defined. Another property of this connection by generalized open mixture arc is that it is an equivalence relation.

**Proposition** **10.** The relation in Definition 2 is an equivalence relation.

**Proof.**  Reflexity and symmetry properties follow from definition. As for the transitivity, consider $p,q\;\mathrm{and}r\in P_{\mu }$ such that $p\left(\lambda \right)=F^{-1}\left((1-\lambda )F(p)+\lambda F(q)\right)\in P_{\mu }$ and $q\left(\beta \right)=F^{-1}\left((1-\beta )F(q)+\beta F(r)\right)\in P_{\mu }$ with λ,β∈[−ε,1+ε] for some ε>0. We can take $p\left(-\varepsilon \right)=F^{-1}\left((1+\varepsilon )F(p)+(-\varepsilon )F(q)\right)$ and $q\left(-\varepsilon \right)=F^{-1}\left((1+\varepsilon )F(q)+(-\varepsilon )F(r)\right)$, and define a probability distribution

$$\begin{array}{cc} p_{1}= & F^{-1}\left(\left(1-\frac{\varepsilon }{1+2\varepsilon }\right)F\left(p\left(-\varepsilon \right)\right)+\frac{\varepsilon }{1+2\varepsilon }F\left(q\left(-\varepsilon \right)\right)\right)\\ = & F^{-1}\left(\frac{{\left(1+\varepsilon \right)}^{2}}{1+2\varepsilon }F\left(p\right)-\frac{\varepsilon^{2}}{1+2\varepsilon }F\left(r\right)\right). \end{array}$$

If we have $p\left(1+\varepsilon \right)=F^{-1}\left((-\varepsilon )F(p)+(1+\varepsilon )F(q)\right)$ and $q\left(1+\varepsilon \right)=F^{-1}\left((-\varepsilon )F(q)+1+\varepsilon )F(r)\right)$, we may define a probability distribution as

$$\begin{array}{cc} p_{2}= & F^{-1}\left(\frac{-\varepsilon }{-1-2\varepsilon }F\left(p\left(1+\varepsilon \right)\right)+\frac{-1-\varepsilon }{-1-2\varepsilon }F\left(q\left(1+\varepsilon \right)\right)\right)\\ = & F^{-1}\left(\frac{-\varepsilon^{2}}{1+2\varepsilon }F\left(p\right)+\frac{{\left(1+\varepsilon \right)}^{2}}{1+2\varepsilon }F\left(r\right)\right). \end{array}$$

The generalized open mixture arc, $r\left(\alpha \right)=F^{-1}\left(\left(1-\alpha \right)F\left(p_{1}\right)+\alpha F\left(p_{2}\right)\right)$, α∈(0,1), connects $r\left(\frac{{\left(1+\varepsilon \right)}^{2}}{2\varepsilon^{2}+2\varepsilon +1}\right)=p$ and $r\left(\frac{\varepsilon^{2}}{2\varepsilon^{2}+2\varepsilon +1}\right)=r$. ☐

## 5. Conclusions

In this work, we have generalized open exponential arc and open mixture arc for probability distributions. Moreover, we ensure that the generalization of open mixture arc is well-defined for deformed exponential strictly convex. From two φ-connected probability distributions $p_{1}$ and $p_{2}$, we can define the generalized parallel transport $\tau_{p_{1},p_{2}}^{\left(1\right)}$ between the tangent spaces $T_{p_{1}}P_{\mu }\;\mathrm{and}\;T_{p_{2}}P_{\mu }$ given by

$$u\mapsto u-\frac{\int_{T}u\varphi^{\prime }\left(\varphi^{-1}\left(p_{2}\right)\right)d\mu }{\int_{T}u_{0}\varphi^{\prime }\left(\varphi^{-1}\left(p_{2}\right)\right)d\mu }u_{0},$$

where $T_{p}P_{\mu }≃B_{c}^{\varphi }$ with p=φ(c). A next step is to find a generalized parallel transport $\tau_{p_{1},p_{2}}^{\left(-1\right)}$ that is dual to $\tau_{p_{1},p_{2}}^{\left(1\right)}$. Another goal is to investigate if the generalized Rényi divergence $D_{\varphi }^{\alpha }\left(\cdot ∥\cdot \right)$ defined in [36] from two probability distributions φ-connected, can be related to the statistical divergence associated with $\left(\tau_{p_{1},p_{2}}^{\left(-1\right)},\tau_{p_{1},p_{2}}^{\left(1\right)},\langle \cdot ,\cdot \rangle \right)$.
